# Seasonal Susceptibility of Winter and Summer Honeybee Workers (*Apis mellifera* L.) to Oral Exposure of Selected Insecticides Under Laboratory Conditions

**DOI:** 10.3390/toxics14080693

**Published:** 2026-08-05

**Authors:** Mohammed A. A. Saad, Verónica Andrade-Yucailla, Ahmed H. A. El-Badry, Aly A. Abd-Ella, Ahmed M. M. Ahmed, Ángel León-Mejía, Víctor González-Rivera, Eslam M. Omar

**Affiliations:** 1 Plant Protection Department, Faculty of Agriculture, Assiut University, Assiut 71526, Egypt; mohamed.abdelbasset@agr.aun.edu.eg (M.A.A.S.); ahmed.helmy3147@gmail.com (A.H.A.E.-B.); aly.abdella@aun.edu.eg (A.A.A.-E.); eslamomar@aun.edu.eg (E.M.O.); 2 Facultad de Ciencias Agrarias, Universidad Estatal Península de Santa Elena, La Libertad 204102, Ecuador; aleon@upse.edu.ec (Á.L.-M.); vgonzalezr@upse.edu.ec (V.G.-R.)

**Keywords:** seasonal toxicity, *Apis mellifera*, insecticides, honeybee workers

## Abstract

To cope with severe seasonal changes in climate and food availability, colonies of *Apis mellifera* L. generate two distinct worker phenotypes during winter and summer. The aim of this research was to evaluate the effect of oral exposure to insecticides on the toxic susceptibility of winter and summer honeybee workers (*Apis mellifera* L.) under laboratory conditions. In this work, under all tested conditions and seasons, emamectin benzoate was the most toxic compound and used as the standard (Toxicity Index = 100) for comparison with other compounds. It had the lowest LC_50_ (Lethal Concentration required to kill 50% of the tested honeybees) values, which decreased to 0.004 mg/L in winter bees after 48 h following oral exposure. Sulfoxaflor had moderate toxicity, and indoxacarb was the least toxic to honeybees. The results also revealed that the toxic effect was time-dependent, with lethal concentrations at 48 h significantly lower than those at 24 h. In addition, seasonal effects play a role; winter honeybees were, overall, more sensitive to the treatments than summer honeybees. Under the conditions of this study, it can be concluded that emamectin benzoate was the most toxic substance and indoxacarb the least toxic. Toxicity increased with exposure time. Winter honeybees showed greater sensitivity than summer honeybees. A field-relevant Hazard Quotient (HQ) analysis further indicated that indoxacarb, despite its lower acute toxicity, exhibited the narrowest margin relative to field-recommended spray concentrations, highlighting the value of incorporating field-relevant exposure metrics alongside LC_50_-based rankings in pollinator risk assessment. It is worth clarifying that this narrower margin reflects indoxacarb’s higher field-recommended application rate relative to its LC_50_, rather than a greater intrinsic toxicity than emamectin benzoate, and the two aspects of risk (intrinsic toxicity vs. use-rate-based field risk) should be interpreted separately.

## 1. Introduction

Honeybees (*Apis mellifera* L.) are among the most important managed pollinators worldwide and contribute substantially to the pollination of agricultural crops and wild flowering plants [1]. The economically valuable products that honeybees produce include pollen, royal jelly, bee venom, beeswax, and honey [1,2]. These products are used in the food, cosmetic, and pharmaceutical industries. Stress from food and water shortages, as well as infestations of internal and external parasites such as Nosema, bacteria, and viruses, is among the factors that affect honeybees. Agrochemical pesticides, which are used on vast areas of farms, fruit orchards, forests, and other ecosystems to control weeds, rodents, insect pests, and plant diseases, have jeopardized beekeeper’s livelihood in recent decades [3]. Exposure to pesticides is one of the potentially dangerous factors influencing bees. Instead of harming bees directly, pesticide exposure usually prevents them from finding supplies of nectar and pollen.

Honeybee colonies are able to tolerate extreme seasonal climatic changes under temperature and food stress by producing two worker phenotypes: summer and winter bees. These two worker groups show significant differences in their lifespan and physiological traits, according to reference [4]. In contrast to summer bees, which have a lifespan of a few weeks (3–6) during the height of the summer season due to high temperatures [5], winter bees, which emerge from the late brood reared by the colony [6], have a much higher life expectancy of 5 to 8 months [7,8]. According to recent research, colony losses mostly occur in winter [9], which may be due to a range of immunological and pathogenic causes that primarily affect winter bees [10]. To adapt to extreme seasonal variations in temperature and nutrient availability, winter and summer bee phenotypes undergo physiological modifications [11]. However, summer bees have been used in the majority of research examining how feeding behavior responds to pesticide exposure [12,13]. Furthermore, summer and winter bees may have different chemical exposure pathways. Winter bees in a cluster would mostly be exposed through contaminated stored food, such as pollen and honey, but summer bees, especially foragers, are more likely to come into contact with pesticides by direct foliar exposure, guttation, and seed-coated dust [14,15].

This is especially true against the background of a worldwide pollinator decline and where exposure to pesticides has been repeatedly pointed out as a major factor for honeybee colony losses in Europe, North America and the Middle East/North Africa (MENA) region [9]. In Egypt and other surrounding Mediterranean countries, the concomitant intensive agricultural pesticide application and the perennial production of honey result in a constant exposure risk that is exacerbated by seasonal physiological susceptibility. By elucidating seasonal differences in toxicity for three common insecticides representing different modes of action, this research lays the groundwork for developing more ecologically relevant pollinator risk assessment frameworks which are directly applicable to pest management policy in agricultural systems where honeybees are vital to both the economy and the environment.

Winter bees differ substantially from their summer counterparts in several physiological dimensions relevant to insecticide susceptibility. First, overwintering adaptations include pronounced fat body hypertrophy and elevated vitellogenin (Vg) protein levels, which serve as nutrient reserves and antioxidants during the winter cluster period [7,8]. Second, immune gene expression is significantly downregulated during winter, with reduced expression of antimicrobial peptide genes and lower hemocyte activity, resulting in compromised immune competence during overwintering [11]. Third, seasonal variation in xenobiotic detoxification capacity has been documented, with winter bees exhibiting lower expression of key detoxification enzymes including cytochrome P450 monooxygenases (e.g., CYP6AS3, CYP9Q1) and glutathione S-transferases compared to summer foragers [16]. Together, these physiological differences provide the biological framework for expecting differential insecticide susceptibility between the two seasonal phenotypes, although the direction and magnitude of this effect for specific insecticides remain insufficiently understood. Emamectin benzoate, a bio-insecticide produced by the bacterium *Streptomyces avermitilis*, interferes with insect nerve signals by binding to glutamate-gated chloride channels and the gamma aminobutyric acid (GABAR) receptor, thereby acting as a chloride channel activator [17]; it is also known for its delayed toxic effects, with mortality increasing progressively over 24–72 h post-exposure due to accumulation and sustained neural disruption [18]. Sulfoxaflor is a systemic insecticide that functions similarly to neonicotinoids, causing severe acute poisoning that damages the neurological system, reduces sucrose response, hinders foraging effectiveness, and, following sublethal exposure, disrupts intestinal metabolism and induces oxidative stress [19]. Indoxacarb is a neurotoxic insecticide that acts as a sodium channel blocker, affecting bee nerve function and causing behavioral changes; although labelled as a reduced-risk pesticide for some insects, it poses a high acute risk to honeybees, particularly if applied directly during foraging [20]. These three compounds were selected because they represent distinct chemical groups and modes of action and are widely used in crop protection programs. Therefore, we hypothesized that winter honeybee workers would exhibit greater susceptibility to oral insecticide exposure than summer workers due to seasonal physiological and metabolic differences, and the aim of this study was to evaluate the seasonal toxicity of emamectin benzoate, sulfoxaflor, and indoxacarb against adult worker honeybees under laboratory oral exposure conditions.

## 2. Materials and Methods

### 2.1. Location and Experimental Management

The experiments were conducted at the Plant Protection Department, Faculty of Agriculture, Assiut University, Assiut, Egypt. Local Carniolan hybrid honeybee workers, *A. mellifera*, were obtained from the Plant Protection Department’s apiary at (27°11′04″ N 31°09′45″ E) during the winter and summer seasons.

Two colonies headed by sister queens that had good hygiene, were free of diseases, and were never exposed to chemicals were used to obtain workers during the two seasons studied. To obtain age-controlled forager workers, brood combs containing sealed brood close to adult emergence were selected and monitored. Newly emerged workers were individually marked and returned to their original colonies. Marked workers were subsequently recaptured at 20 days of age, corresponding to the typical onset of foraging activity in honeybees. Only marked forager workers were used in all bioassays. Two sister-queen colonies were used per season [16,21]. At least 200 forager bees per treatment group per season were used for all replicates, which is enough to provide sufficient statistical power for Probit analysis. In total, each insecticide–season combination involved seven test concentrations plus a control, each concentration comprising three cages of ten bees per replicate (30 bees/concentration/replicate) across two independent replicates (60 bees per concentration), yielding approximately 480 bees per insecticide per season (420 bees across the seven treated concentrations plus 60 control bees), consistent with the ≥200-bee minimum stated above.

Insecticides: Three insecticides, belonging to different chemical groups and having different modes of action, were chosen because of their extensive use in the Egyptian agroecosystem for the control of various agricultural insect pests. The insecticides were obtained from Central Agricultural Pesticide Laboratory (CAPL) in Dokki, Giza, Egypt. The compounds tested were emamectin benzoate (Basha 1.9% EC, Avermectin group, IRAC Group 6), sulfoxaflor (Isoclast 11.7% SC, Sulfoximine group, IRAC Group 4C) and indoxacarb (Easo Plus 30% WG, Oxadiazine group, IRAC Group 22A). All concentrations in the bioassays are given as the amount of active ingredient in mg per L (mg a.i./L).

**Figure 1:**
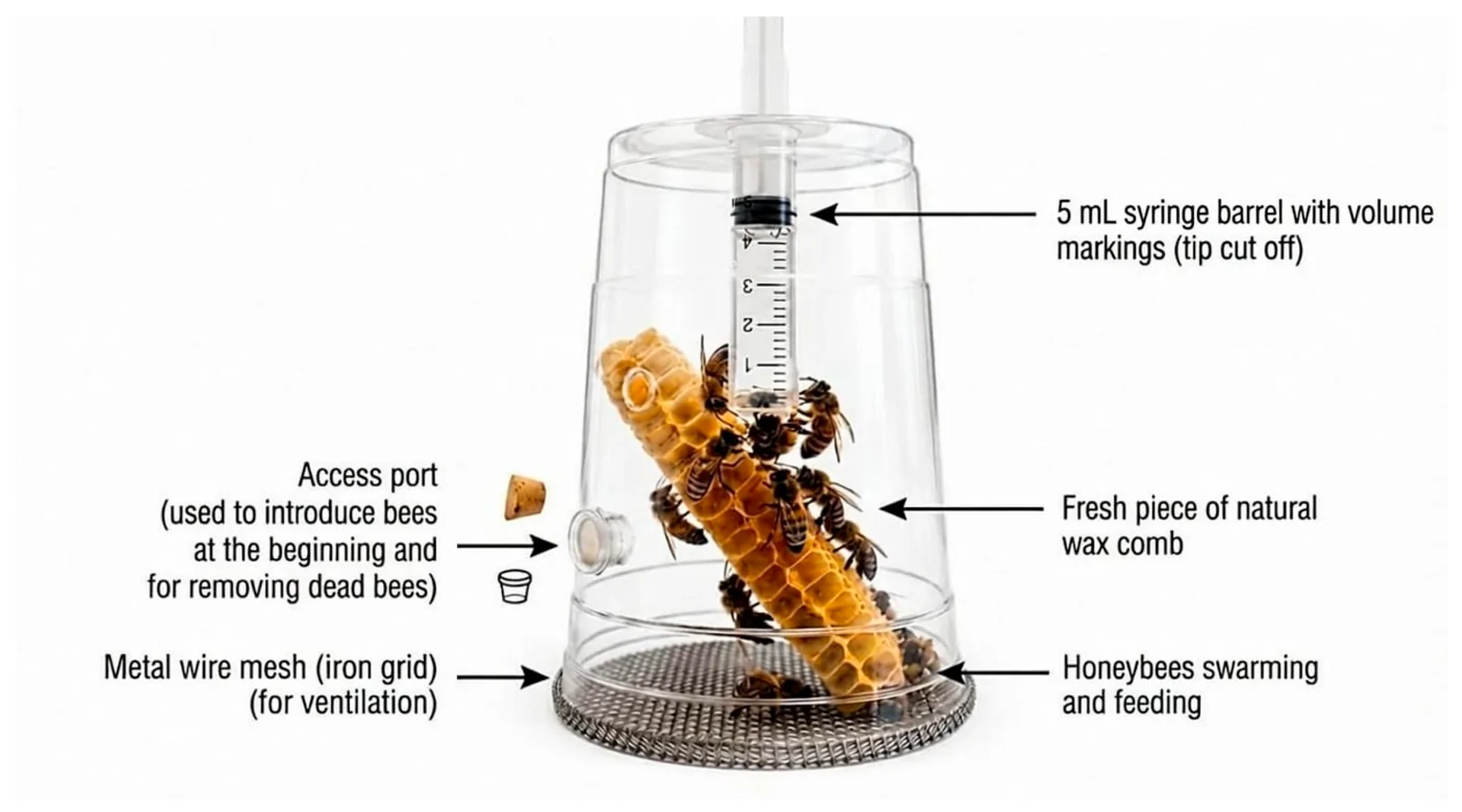
Plastic experimental cage used for oral toxicity bioassays on adult honeybee workers under laboratory conditions.

Experimental cages: According to [22], plastic cages (Figure 1) were used in the experiment. Each cage is made of a clear plastic cup with two open holes: one in the side of the cup to remove dead bees and another on the base to introduce the sugar syrup solution using a 5 mL syringe. A piece of organic wax comb was placed inside the cup to stimulate the honeybees’ natural state. An iron grid that allowed air to get through was placed over the cup’s nozzle [21].

The following environmental conditions were maintained throughout all bioassays: temperature 34 ± 1 °C for summer bioassays and 34 ± 1 °C for winter bioassays; relative humidity 60 ± 5%; constant darkness (DD photoperiod) in accordance with [21]. Each plastic cage (clear cup, approximately 250 mL) contained 10 bees, with 3 cages per concentration (*n* = 30 bees per concentration per replicate). Prior to bioassay initiation, bees starved for 2 h to standardize hunger levels. Treated sucrose syrup (1:1 *w*/*v*) was offered ad libitum via a 5 mL syringe through the top hole for the first 4 h; thereafter, untreated syrup was provided for the remainder of the 24 and 48 h observation periods.

Toxicity bioassay: A mixed technique derived from the protocols of the Guideline [23,24] and [25] were used for two experimental steps. Serial dilutions (1:10) of pesticide stock (1000 mg a.i./L) in distilled water were used for preliminary testing. To determine the dose that results in 10–90% mortality, seven concentrations were prepared in descending order. This common range-finding series was applied to all three insecticides to bracket the concentration range producing 10–90% mortality: 1000, 100, 10, 1, 0.1, 0.01, 0.001, and 0.0001 mg a.i./L. Based on the results of this preliminary stage, insecticide-specific definitive concentration series were then selected for the main bioassays, since the concentration range needed to bracket the LC_10_–LC_90_ mortality window differed substantially among the three compounds according to their intrinsic potency (e.g., for summer bees: sulfoxaflor, 0.1–1.0 mg a.i./L; indoxacarb, 3–20 mg a.i./L; and emamectin benzoate, 0.008–0.5 mg a.i./L; four–five concentrations plus a control per insecticide, as reported in Table 1, Table 2, Table 3 and Table 4). The same insecticide-specific definitive concentration series (sulfoxaflor, 0.1–1.0 mg a.i./L; indoxacarb, 3–20 mg a.i./L; emamectin benzoate, 0.008–0.5 mg a.i./L; four–five concentrations plus a control per insecticide) was used for both the winter and summer bioassays. The concentration unit was corrected from ng a.i./L to mg a.i./L, consistent with the LC_50_/LC_90_ values (mg/L) reported in Table 1, Table 2, Table 3 and Table 4 and with the field-recommended concentrations (mg a.i./L) used in the Hazard Quotient calculation. No individual test concentrations were excluded from the Probit analyses (e.g., for showing 0% or 100% mortality); only entire replicates failing the ≤10% control-mortality criterion were excluded, as stated above.

Only sugar syrup (*v*/*v* 1 sugar:1 water) was used as the control treatment. The percentage of bees that died was noted after 24 and 48 h. Each insecticide toxicity test was conducted twice, and Abbott’s formula was used to adjust the fatality rate, with each replicate comprising three cages of ten bees per concentration (*n* = 30 bees per concentration per replicate). Each experiment was independently repeated in two separate biological replicates conducted on different dates, ensuring reproducibility. Only replicates with control mortality below 10% were retained for analysis, in accordance with OECD guideline 213. Insecticides LC_50_ and slope values were calculated using the Probit regression analysis tool and reported in mg/L. The toxicity index was determined using Sun’s formula [26,27]:

Toxicity index = (The LC_50_ value of the most toxic insecticide/the LC_50_ value of the tested insecticide) × 100

Seasonal susceptibility differences between winter and summer honeybees were determined by calculating the Sensitivity Ratio Toxicity index = (The LC_50_ value of the most toxic insecticide/the LC_50_ value of the tested insecticide) × 100

Toxicity index = (The LC_50_ value of the most toxic insecticide/the LC_50_ value of the tested insecticide) × 100

Sensitivity Ratio = (LC_50_ of the less sensitive season/LC_50_ of the more sensitive season)

### 2.2. Statistical Analysis

According to [28], the LC_50_ and LC_90_ (Lethal Concentration required to kill 90% of the tested honeybees), 95% fiducial limits (FL), and slope values for these pesticides were obtained by applying the Probit analysis to the data using the SPSS program version 26 (IBM Corp., Armonk, NY, USA). The non-overlap between the 95% confidence intervals of two LC_50_ values, reported in mg/L, provided the basis for a significant level of mean separation (*p* < 0.05). The Sensitivity Ratio (SR) was calculated as SR = LC_50_ of the less sensitive season/LC_50_ of the more sensitive season [27]; SR values > 1 indicate greater susceptibility of the more sensitive phenotype, with larger values reflecting more pronounced seasonal differences. The Toxicity Increase Factor (TIF) was calculated as TIF = LC_50_ at 24 h/LC_50_ at 48 h; values > 1 indicate a time-dependent increase in toxicity, with higher values reflecting greater sensitivity to prolonged exposure. Regarding statistical comparisons, LC_50_ and LC_90_ values were considered significantly different when their 95% fiducial limits (confidence intervals) did not overlap, a criterion widely accepted in Probit-based honeybee toxicology studies and consistent with OECD guideline 213 recommendations. The SR and TIF values serve as descriptive indices to quantify the magnitude of seasonal and temporal differences, respectively; SR values substantially greater than 1.0 indicate biologically meaningful seasonal shifts in susceptibility, while TIF values substantially greater than 1.0 indicate pronounced time-dependent toxicity increases.

### 2.3. Field-Relevant Hazard Quotient (HQ)

To contextualize the laboratory-derived toxicity estimates within a realistic field exposure scenario, a Hazard Quotient (HQ) was calculated for each insecticide, following the conceptual framework of pesticide risk assessment for pollinators [29,30]: HQ = Field-recommended spray concentration (mg a.i./L)/LC_50_ (mg a.i./L). The field-recommended spray concentration of each commercial formulation was derived from the manufacturer’s label dosage and the corresponding official Egyptian field recommendations for the target pests of each crop: Basha 1.9% EC (emamectin benzoate) at 1.25 mL/L water, equivalent to 23.75 mg a.i./L; Isoclast 11.7% SC (sulfoxaflor) at 0.50 mL/L water, equivalent to 58.5 mg a.i./L; and Easo Plus 30% WG (indoxacarb) at 0.3 g/L water, equivalent to 90 mg a.i./L. Higher HQ values indicate a wider margin between the field-applied concentration and the laboratory-derived lethal threshold, providing a conservative, worst-case approximation of bee exposure to contaminated nectar, guttation droplets, or direct spray residues near treated fields. This HQ approach is intended as a simplified, screening-level index for comparative purposes and does not constitute a comprehensive field risk assessment, which would additionally require data on residue decay over time, forager behavior, and colony-level exposure dynamics.

## 3. Results

The toxicity of three insecticides (Emamectin benzoate, Sulfoxaflor, and Indoxacarb) was evaluated at 24 and 48 h after treatment in winter and summer honeybees. The toxicity index was determined using Emamectin benzoate as the reference (100), and toxicity was evaluated based on the Median Lethal Concentrations (LC_50_ and LC_90_).

### 3.1. Toxicity Evaluation for Winter Honeybees

#### 3.1.1. Exposure of Winter Honeybees to Insecticides for 24 h

Data in Table 1 showed that emamectin benzoate had the highest toxicity with an LC_50_ of 0.035 mg/L and an LC_90_ of 0.338 mg/L at 24 h post-treatment. Sulfoxaflor followed with an LC_50_ of 1.101 mg/L and a toxicity index of 3.18, while indoxacarb was the least toxic with an LC_50_ of 5.720 mg/L and a toxicity index of 0.61.

#### 3.1.2. Insecticides Toxicity on the Winter Honeybee Workers After 48 h

At 48 h post-treatment, the toxicity of all substances increased considerably. Among all tested insecticides, Emamectin benzoate was the most toxic, with an LC_50_ of 0.004 mg/L (Table 2). Sulfoxaflor exhibited an intermediate toxicity with an LC_50_ = 0.187 mg/L. Indoxacarb, on the other hand, was the least toxic with a value of 0.725 mg/L. The slope for Indoxacarb increased from 1.127 to 2.144, indicating that the rate of increase in mortality over time was faster at higher concentrations. Mortality across all treatments was consistently higher at 48 h than at 24 h, which may suggest a delayed mode of action or the accumulation of other physiological damage over time.

### 3.2. Toxicity Assessment for Summer Honeybees

#### 3.2.1. Insecticides Toxicity on Summer Honeybee Workers After 24 h

The results in Table 3 showed that, for the summer honeybee worker population at 24 h, the toxicity order was Emamectin benzoate > Sulfoxaflor > Indoxacarb; Emamectin benzoate exhibited the highest toxicity (LC_50_: 0.036 mg/L). Sulfoxaflor had a relatively high LC_90_ toxicity index of 51.56, with an LC_50_ of 0.958 mg/L. Indoxacarb was the least susceptible in the series (LC_50_: 13.356 mg/L).

#### 3.2.2. Insecticides Toxicity on Summer Honeybee Workers After 48 h

The toxicity of the insecticides studied for summer honeybees was ranked after 48 h exposure, in order of decreasing LC_50_ and LC_90_ values (Table 4). Among the compounds tested, Emamectin benzoate was the most toxic, with the lowest LC_50_ value (0.009 mg/L) and was therefore considered the standard compound, with a toxicity index of 100 for both LC_50_ and LC_90_. Sulfoxaflor was moderately toxic among the insecticides, with an LC_50_ value of 0.571 mg/L and an LC_90_ of 1.380 mg/L. The associated toxicity indices were 1.58 and 9.71 (LC_50_ and LC_90_), indicating lower toxicity than Emamectin benzoate. Of the three insecticides tested, Indoxacarb was the least toxic, with LC_50_ and LC_90_ values of 4.545 mg/L and 10.417 mg/L, respectively. High slope estimates for Sulfoxaflor and Indoxacarb (>3.0) 48 h. post-treatment indicates steep increases in the number of dead honeybees, with only small increments in concentration.

**Figure 2:**
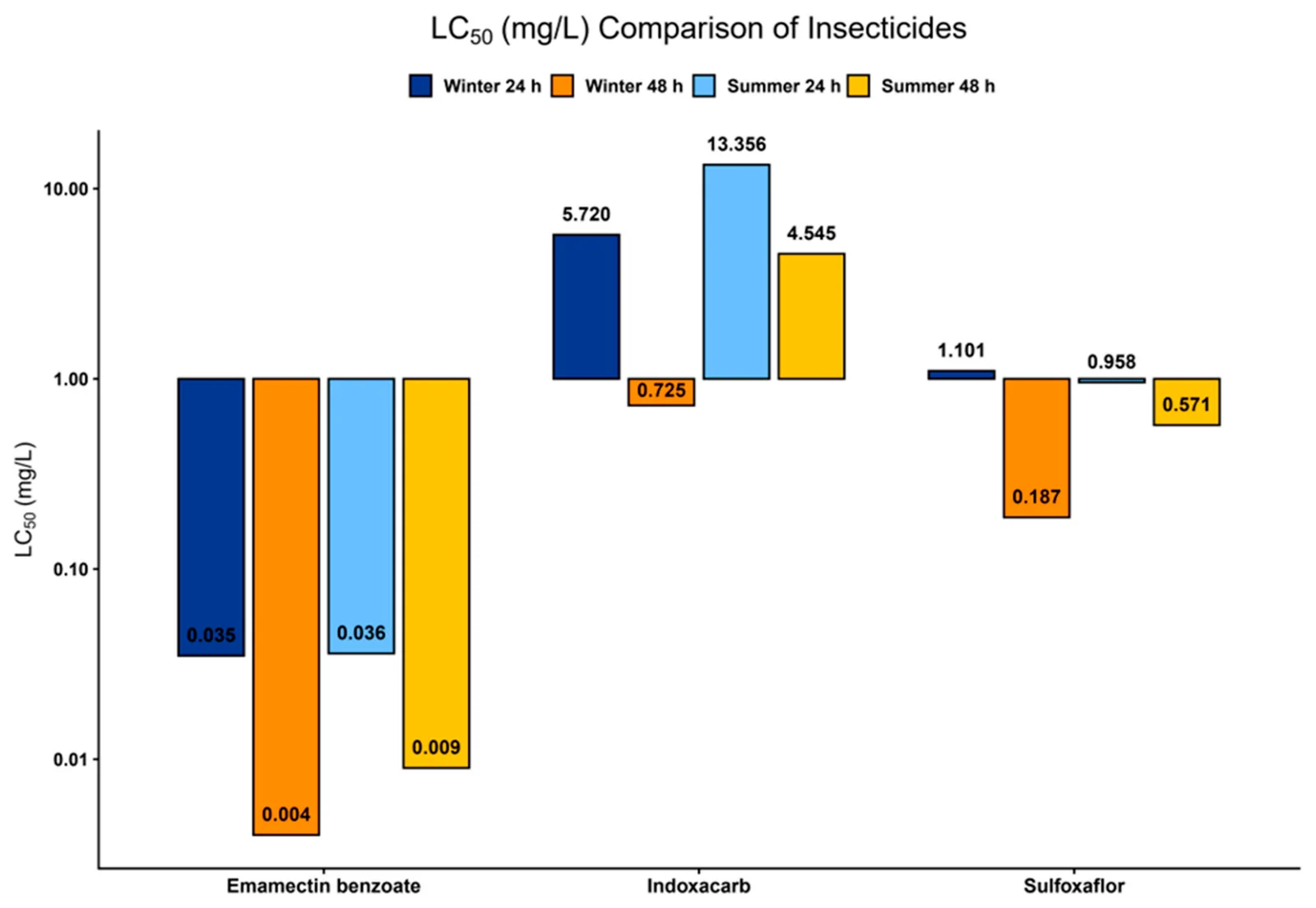
Comparative LC_50_ values of selected insecticides against winter and summer honeybee workers after 24 and 48 h of oral exposure.

The LC_50_ values of the tested insecticides against winter and summer workers calculated at 24 and 48 h following oral intake exposure are shown in Figure 2. Emamectin benzoate exhibited the greatest toxicity in both seasons, and sulfoxaflor showed the highest toxicity among the novel insecticides, followed by indoxacarb, which was the least toxic compound. LC_50_ values for all treatments significantly declined to 48 h as compared to 24 h, which suggests a pronounced time-dependent increase in toxicity. Winter honeybee workers tend to be more sensitive than summer workers particularly after 48 h exposure; this supports the idea that the seasonal physiological status of the insects tested influenced their susceptibility to the insecticides investigated.

### 3.3. Comparative Sensitivity Ratio (SR) Between Winter and Summer Honeybees

**Figure 3:**
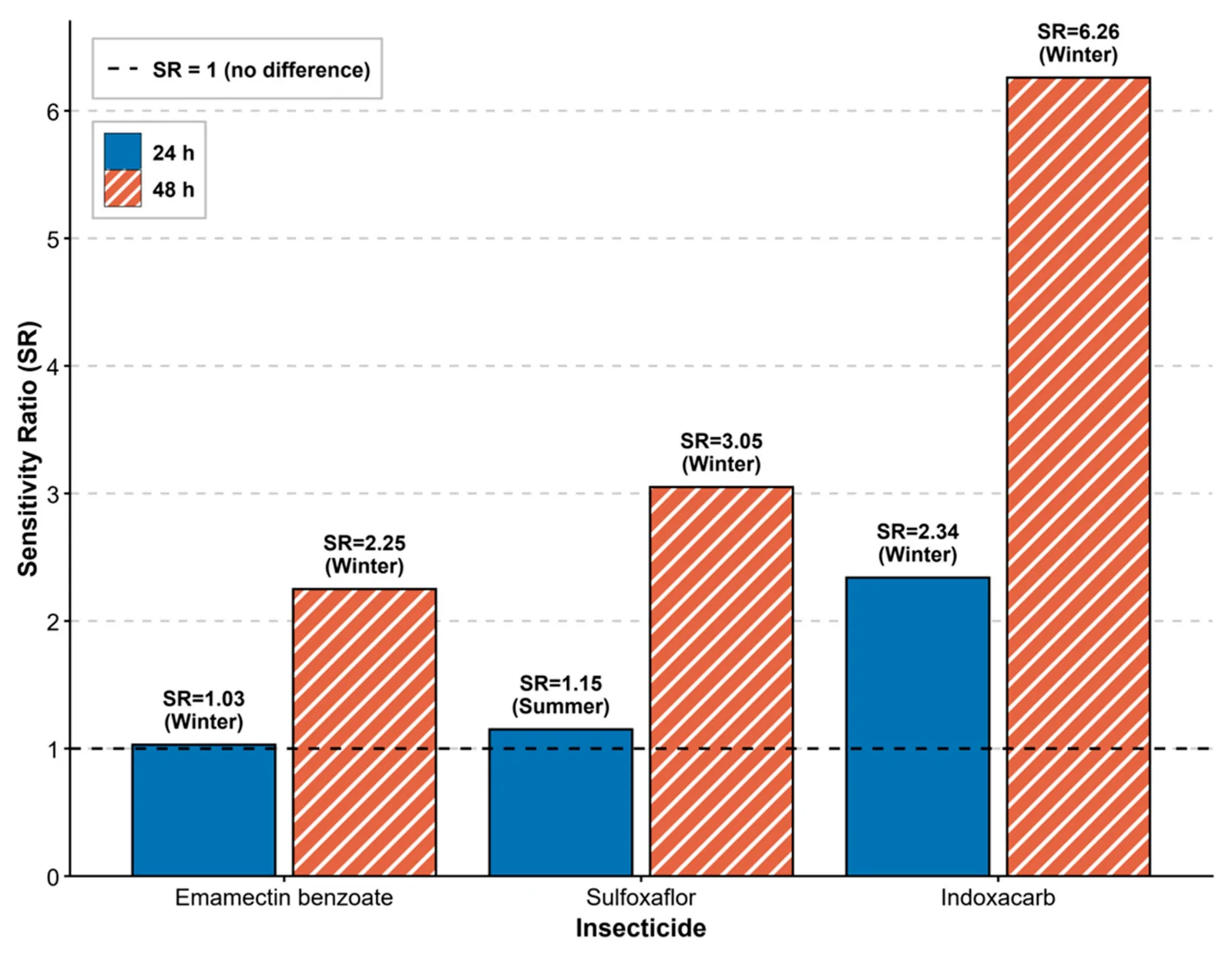
Comparative sensitivity ratio (SR) between winter and summer honeybee workers after 24 and 48 h of oral exposure to the tested insecticides.

The comparative sensitivity ratio (SR) between winter and summer honeybees is reported for each insecticide exposure period (Table 5 and Figure 3). Winter honeybees were slightly more sensitive than summer honeybees at 24 h (SR = 1.03) for Emamectin benzoate, and a higher difference was detected at 48 h (SR = 2.25). In the case of sulfoxaflor, a slightly higher sensitivity of summer honeybees at 24 h (SR = 1.15) was found, at 48 h, higher sensitivity was detected in winter honeybees, together with a significant increase in the SR (3.05). For Indoxacarb, winter honeybees were significantly more sensitive than summer honeybees at both exposure times, with SR values of 2.34 and 6.26 at 24 h and 48 h, respectively, indicating a marked seasonal variation in response at longer exposure times. Overall, winter honeybees tend to be more sensitive to most treatments, especially at 48 h exposure.

### 3.4. Toxicity Increase Factor (TIF) over Exposure Duration 24 h vs. 48 h

**Figure 4:**
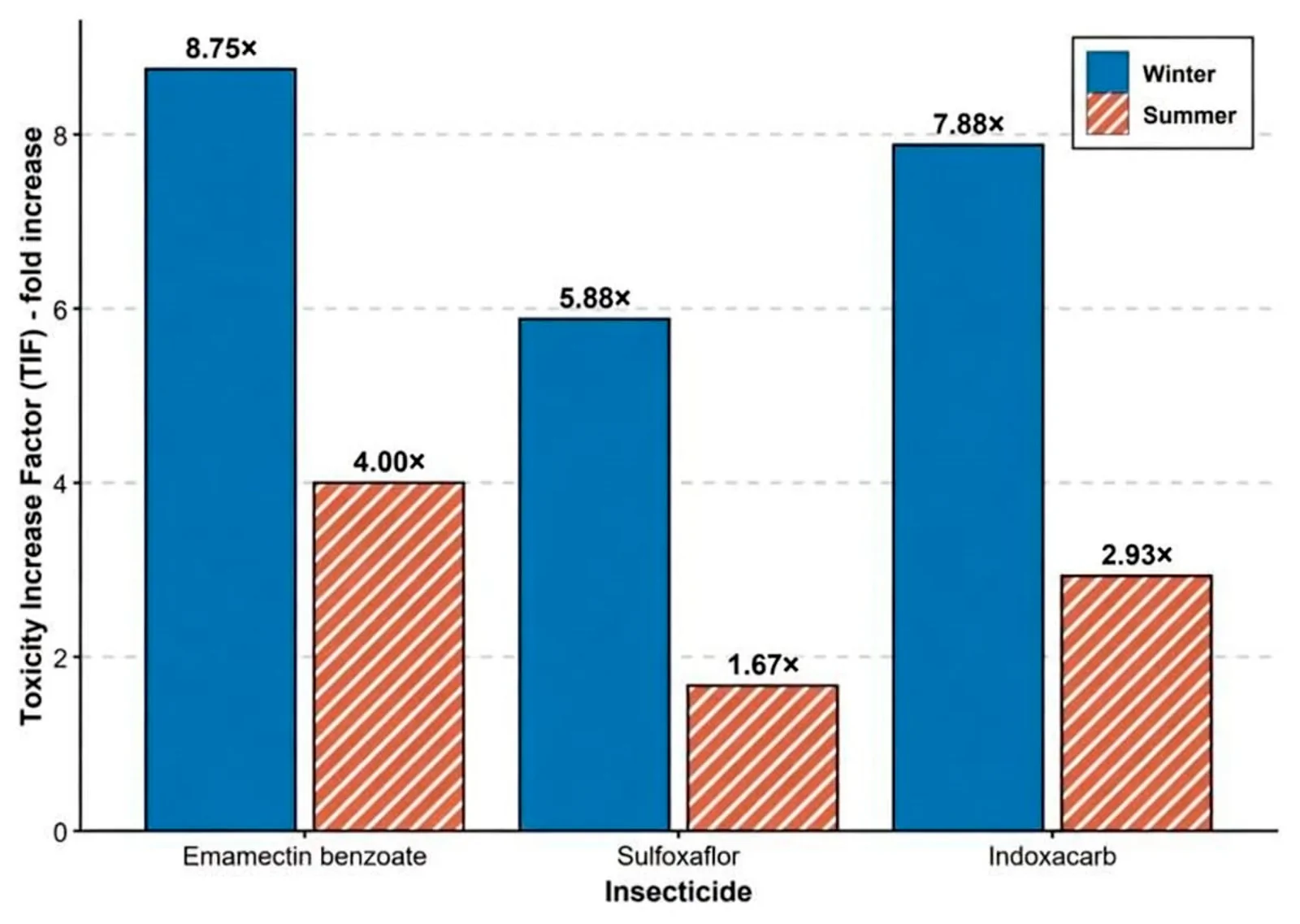
Toxicity Increase Factor (TIF) of emamectin benzoate, sulfoxaflor, and indoxacarb against winter and summer honeybee workers after 24 and 48 h of oral exposure.

The Toxicity Increase Factor (TIF) showed a distinct rising trend with exposure duration for all insecticides (Table 6 and Figure 4). In winter honeybees, emamectin benzoate had the highest TIF (8.75×), followed by indoxacarb (7.88×) and sulfoxaflor (5.88×), indicating a significant time-dependent increase in toxicity. Emamectin benzoate also had the highest TIF (4.00×) in summer honeybees, while indoxacarb and sulfoxaflor had smaller increases of 2.93× and 1.67×, respectively. In general, all tested insecticides showed increased toxicity after 48 h compared to 24 h, and winter honeybees were more sensitive to time-dependent increases in toxicity than summer honeybees.

**Figure 5:**
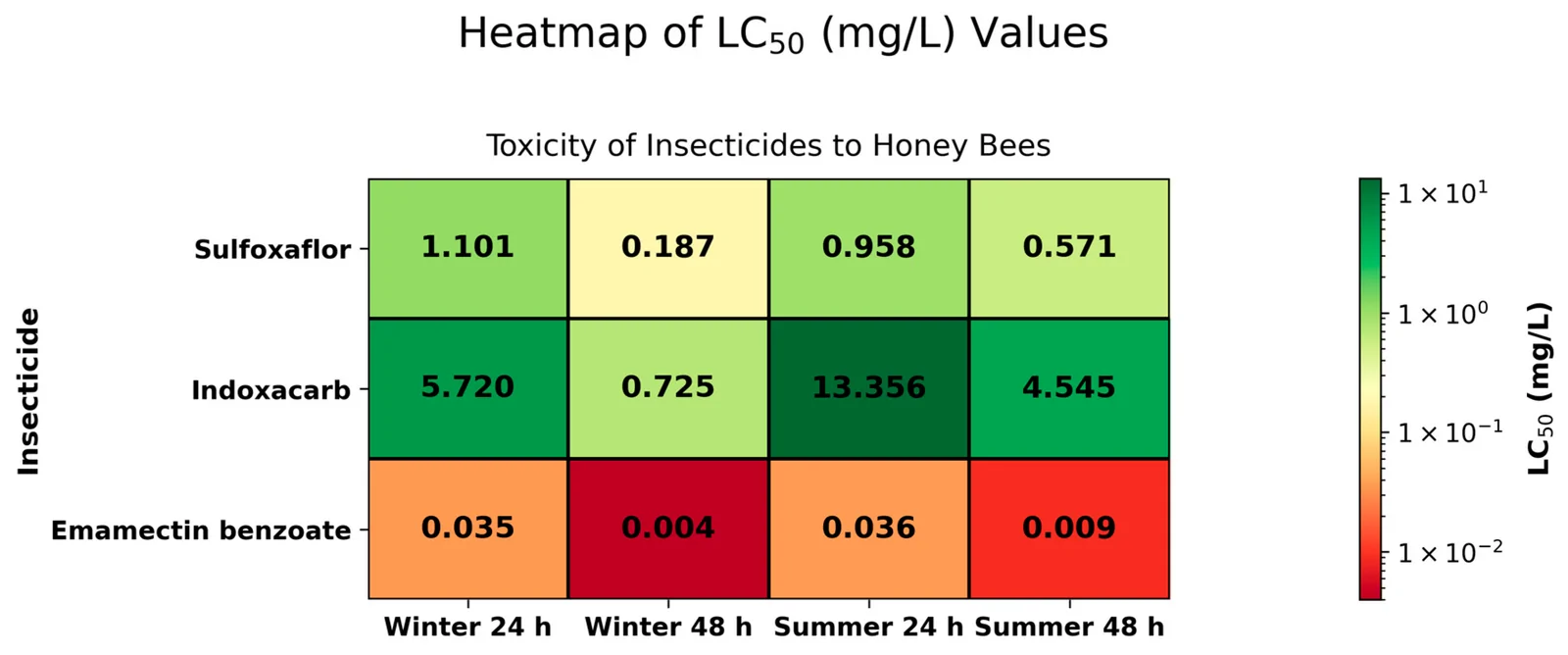
Heatmap of LC_50_ (mg/L) values for emamectin benzoate, sulfoxaflor, and indoxacarb against winter and summer honeybee workers after 24 and 48 h of oral exposure.

Three insecticides, Emamectin benzoate, Sulfoxaflor and Indoxacarb were tested against winter and summer honeybee workers through oral exposure after 24 and 48 h, shown in the heatmap of LC_50_ values (Figure 5). Degree of toxicity has been indicated as a color scale, where red represents low values of LC_50_ (high toxicity) and green represents high values of LC_50_ (low toxicity). The most toxic compound was emamectin benzoate under all tested circumstances, followed by Sulfoxaflor, and indoxacarb was the least toxic. Moreover, the toxicity tended to increase with time of exposure, as indicated by the decrease in LC_50_ values from 24 to 48 h for all insecticides. The heatmap also reveals seasonal differences in susceptibility, but the response pattern varied among insecticides and exposure time.

### 3.5. Field-Relevant Hazard Quotient (HQ)

To evaluate the practical field relevance of the laboratory toxicity data, the Hazard Quotient (HQ) was calculated for each insecticide by comparing the field-recommended spray concentration with the experimentally derived LC_50_ values (Table 7). Emamectin benzoate exhibited the highest HQ values among the three compounds, ranging from 659.72 (summer, 24 h) to 5937.50 (winter, 48 h), reflecting an exceptionally wide margin between its lethal threshold and the concentration applied in the field. Sulfoxaflor showed intermediate HQ values, ranging from 53.13 (winter, 24 h) to 312.83 (winter, 48 h). Indoxacarb, despite recording the highest (least toxic) LC_50_ values among the three insecticides, displayed the narrowest HQ range, from 6.74 (summer, 24 h) to 124.14 (winter, 48 h), indicating that its margin between the recommended field concentration and the lethal threshold is considerably smaller than would be inferred from its LC_50_ ranking alone.

## 4. Discussion

The present study demonstrates that emamectin benzoate exhibited the highest toxicity to worker honeybees (*Apis mellifera* L.) in both winter and summer phenotypes, recording the lowest LC_50_ values of 0.004 mg/L in winter honeybee workers and 0.009 mg/L in summer honeybee workers at 48 h post-treatment, compared to sulfoxaflor (moderately toxic 0.187 and 0.571 mg/L in winter and summer after 48 h post treatment, respectively), and indoxacarb was the least toxic. This is consistent with emamectin benzoate’s mechanism of action described in Section 1 [17]. The relatively wide confidence interval associated with the LC_90_ estimate for indoxacarb (Table 1) may reflect increased variability in mortality responses at higher concentration levels, as well as the lower precision typically associated with estimating upper lethal thresholds from probit regression analyses. Consequently, the LC_90_ estimate should be interpreted with caution. More broadly, several LC_90_ estimates reported in this study (Table 1, Table 2, Table 3 and Table 4) show comparatively wide 95% fiducial limits, which is a common feature of upper-percentile Probit estimates in acute bioassays; these values should therefore be regarded as indicative rather than precise, and cross-compound or cross-season comparisons based on LC_90_ alone should be made with appropriate caution. However, this uncertainty does not affect the overall conclusion regarding the comparatively high toxicity of indoxacarb or the pronounced seasonal differences in susceptibility observed between winter and summer honeybee workers.

To better interpret the observed toxicity ranking, it is important to consider the interaction between each insecticide’s mode of action, physicochemical properties, and the physiological characteristics of honeybees. The very low LC_50_ values observed for emamectin benzoate may reflect the combined effects of its high intrinsic potency, favorable physicochemical properties that facilitate uptake, and its interaction with glutamate-gated chloride channels. Although these toxicokinetic processes were not directly measured in the present study, this interpretation is consistent with the known mode of action of avermectin compounds [17]. Acting as an agonist of the nicotinic acetylcholine receptor, but binding this target with high affinity, Sulfoxaflor also shows intermediate LC50 values relative to emamectin benzoate and indoxacarb. The roughly 50- to 60-fold higher LC50 of sulfoxaflor compared with emamectin benzoate at 48 h suggests that factors beyond receptor affinity, including toxicokinetic and detoxification processes, contribute to its lower acute toxicity relative to emamectin benzoate, and these differences in toxicity possibly indicate a more efficient detoxification route, most likely cytochrome P450-mediated oxidation, which seems to agree with some evidence of seasonal fluctuation among detoxification enzymes in honeybees [16,18]. The relatively low acute toxicity of indoxacarb, in turn, is consistent with its nature as a pro-insecticide: the parent compound has little activity at the voltage-gated sodium channel and its toxicity is a function of bioactivation by esterase and amidase enzymes to the metabolite DCJW [20], and therefore the rate-limiting step to lethality is metabolic conversion rather than direct interaction with its receptor. This gives a plausible biochemical rationale for the finding that indoxacarb was far less acutely toxic than the other two compounds, even though all three compounds ultimately exert their effects on the nervous system. Several methodological choices made in this study warrant brief justification here. Age was standardized by monitoring brood combs, individually marking newly emerged workers, and recapturing them as foragers at 20 days of age so that all experimental individuals were of known age; this approach minimizes age-related physiological variability and increases the consistency and reliability of the toxicity assessments relative to bioassays performed on foragers of unknown or highly variable age. Two sister-queen colonies were used per season as a deliberate design to minimize inter-colony genetic variability so that observed differences in susceptibility could be attributed primarily to seasonal physiological state rather than genetic background [16,21]; although OECD/EPPO protocols do not specify a minimum number of colonies, genetic homogeneity and an adequate number of bees per replicate are emphasized as the more critical considerations. The two-replicate design adopted here is consistent with published honeybee oral toxicity studies and provides adequate biological replication for Probit-based LC_50_ estimation [25]. Finally, reliance on non-overlapping 95% fiducial limits, rather than additional formal interaction tests, follows a conservative and widely accepted approach for pairwise comparison of Probit-derived lethal concentration estimates [28].

These findings corroborate prior research, such as that of [2], who reported similar toxic effects of various pesticides on honeybees under laboratory conditions. The most toxic pesticide during the exposure period 24, 48 and 72 h was emamectin benzoate with LC_50_ values (0.247, 0.047 and 0.020 mg/L) and with LC_90_ values (5.752, 0.302 and 0.072 mg/L), respectively. The most to least toxic pesticides were arranged as follows: Emamectin benzoate > imidacloprid > chlorpyrifos > indoxacarb > lambda-cyhalothrin > thiophanate-methyl > glyphosate.

Emamectin benzoate exhibited higher toxicity as exposure time increased, with values significantly decreasing between 24 and 48 h. The toxicity of EB in winter bees increased by up to 8.75-fold between 24 and 48 h, suggesting delayed physiological damage or slowed metabolic breakdown. This increased mortality likely reflects a combination of prolonged neural disruption and impaired detoxification capacity, as emamectin benzoate inhibits glutamate-gated chloride channels leading to sustained excitatory signaling (note: no immune biomarkers were measured in this study; immune effects discussed elsewhere are based on the published literature). Some studies suggest that the high toxicity of Emamectin benzoate is due to a combination of efficient cuticular and gut penetration and low detoxification rates, allowing the toxin to accumulate and cause damage over an extended period [31]. This is consistent with the marked increase in toxicity observed between 24 and 48 h in winter bees, suggesting that delayed toxicodynamic responses and cumulative physiological effects may contribute to the progressive increase in mortality.

Seasonal differences showed that winter bees were generally more susceptible than summer bees, with a sensitivity ratio (SR) > 1 in most cases, up to 6.26 for indoxacarb at 48 h. This pattern is consistent with the seasonal physiological and exposure-pathway differences between winter and summer bees already outlined in Section 1 (e.g., longer winter lifespan, reliance on stored pollen/honey, and reduced winter immune and detoxification capacity [4,7,8,11,16]). Notably, summer bees showed slightly higher sensitivity to sulfoxaflor at 24 h (SR = 1.15), likely due to greater direct foraging exposure. These results are consistent with the known properties of sulfoxaflor, which, akin to neonicotinoids, induces neurological damage in adult bees, as observed in the present study. Taken together, the SR values obtained across the three insecticides (1.03–2.25 for emamectin benzoate, 1.15–3.05 for sulfoxaflor, and 2.34–6.26 for indoxacarb across the 24–48 h window) reveal a consistent pattern in which seasonal susceptibility differences widen substantially between 24 and 48 h for every compound, reinforcing that time-dependent processes, rather than an immediate difference in target-site sensitivity, underlie the greater vulnerability of winter bees. It should be noted that effects on larvae and under chronic, environmentally relevant exposure have also been reported elsewhere in the literature [19], but these routes were not examined in the present acute, adult-only laboratory bioassay.

The non-uniform magnitude of seasonal susceptibility differences among the three insecticides warrants mechanistic consideration. Emamectin benzoate exhibited a relatively small seasonal sensitivity ratio at 24 h (SR = 1.03) but a larger difference at 48 h (SR = 2.25). This pattern suggests that seasonal variation may be associated more with differences in toxicokinetic processes and time-dependent accumulation than with immediate differences in neural sensitivity. Previous studies have reported seasonal variation in honeybee physiology and xenobiotic metabolism, including changes in detoxification-related enzyme systems [16,18], which may contribute to the greater susceptibility of winter bees. In contrast, indoxacarb exhibited the largest seasonal sensitivity ratios (2.34 at 24 h and 6.26 at 48 h). Because indoxacarb functions as a pro-insecticide that requires metabolic bioactivation to its toxic metabolite, decarbomethoxylated indoxacarb (DCJW) [20], seasonal differences in metabolic activity may influence both detoxification and bioactivation processes. Although these mechanisms were not directly examined in the present study, altered enzymatic activity in winter bees could contribute to the pronounced seasonal differences observed for this compound. Sulfoxaflor, a nicotinic acetylcholine receptor (nAChR) agonist with a mode of action similar to neonicotinoids [19], displayed a different pattern. Summer bees were slightly more sensitive at 24 h (SR = 1.15), whereas winter bees became substantially more susceptible after 48 h (SR = 3.05). This shift may reflect differences in physiological state, metabolic capacity, and cumulative toxic effects over time. Collectively, these insecticide-specific patterns indicate that seasonal susceptibility is not a uniform phenomenon but is likely influenced by interactions among insecticide mode of action, metabolic processing, and seasonal physiological characteristics of honeybee workers [16,18].

These seasonal fluctuations can also be interpreted in view of the unique physiological phenotype of diutinus (winter) honeybees. In contrast to the short-lived summer foragers, winter bees present high levels of vitellogenin, larger fat body stores, and a metabolically quiescent physiological state that supports colony survival through the winter, yet this phenotype has also been linked with diminished constitutive activities of several detoxification enzyme systems, such as particular isoforms of cytochrome P450 and glutathione S-transferase, when compared to active foraging summer bees [16,18]. Compromised basal detoxification capacity, combined with the toxicokinetic and toxicodynamic differences among the three insecticides described above, provides a plausible physiological explanation for the consistent higher vulnerability of winter bees reported in this study, and it provides a plausible explanation for the compound-specific differences in sensitivity ratios based on one common seasonal mechanism rather than one separate explanation for each compound. In Egypt’s agro-ecosystem, where these insecticides target key pests, findings underscore risks to pollinators, especially winter bees that depend on stored forage. Recommendations include off-season applications, buffer zones around apiaries, and integrated pest management to safeguard bees, which contribute substantially to the pollination of a large proportion of flowering plants and many agricultural crops. While the present study focused exclusively on acute mortality endpoints (LC_50_ and LC_90_), sublethal effects of these insecticides also warrant consideration. The following discussion is based on evidence from the published literature; no sublethal data was collected in this study. It has been shown that emamectin benzoate at sublethal concentrations can interfere with learning and memory of *Apis mellifera* through blocking glutamate-gated chloride channels in the mushroom bodies and decrease foraging efficiency and hive-return rates [31]. Sulfoxaflor similarly reduces sucrose responsiveness, impacts navigation, and depresses immune response in worker bees at sublethal concentrations [19]. Indoxacarb, along with being the least acutely toxic in this study, might also induce a sublethal neurological effect through the sodium channel blockage with possible disruption of thermoregulation and winter cluster dynamics in winter bees [20]. These sublethal routes are especially important to consider for winter bees since their survival over the course of 5–8 months necessitates a lengthy accumulation of feeding on contaminated stored pollen and honey. The pronounced seasonal differences in LC_50_ values observed here, most notably the increased susceptibility of winter bees by a factor of 6.26 to indoxacarb at 48 h, indicate that the existing pesticide risk assessment schemes, based primarily on data from summer foragers, may significantly over- or under-estimate the risk posed to overwintering colonies.

Beyond the acute toxicity rankings described above, translating LC_50_ values into field-relevant hazard indices provides additional insight into the comparative risk posed by each insecticide under realistic agricultural use. The Hazard Quotient (HQ) analysis (Table 7) revealed a markedly different risk hierarchy than that suggested by LC_50_ alone: although indoxacarb was consistently the least acutely toxic compound on a concentration basis, its narrow margin between the field-recommended spray concentration and the experimental LC_50_ (HQ as low as 6.74–124.14) indicates that, at recommended field rates, the gap between an environmentally realistic exposure concentration and a lethal concentration is considerably smaller for indoxacarb than for emamectin benzoate, whose extremely high HQ values (up to 5937.50) reflect a substantial buffer before field concentrations approach lethal thresholds despite its high intrinsic potency. This finding illustrates that intrinsic toxicity (LC_50_) and field-relevant hazard are not always concordant and that risk assessments relying solely on LC_50_ rankings may misrepresent the comparative field risk among insecticides with different recommended application rates [29,30]. These results reinforce the value of incorporating both intrinsic toxicity and use-pattern-specific exposure metrics into pollinator risk assessment, particularly for compounds such as indoxacarb that may appear comparatively safe based on LC_50_ alone but carry a narrower margin of safety under actual field conditions. It should be emphasized that the HQ values reported here are a simplified, screening-level comparison intended to flag compounds warranting closer scrutiny, rather than a full field-risk assessment; they do not, for example, account for residue degradation, realistic bee foraging exposure patterns, or colony-level dynamics.

It is also important to interpret these findings within the broader context of the multiple, potentially interacting stressors that honeybee colonies experience under field conditions. *Varroa destructor* parasitism, *Nosema* infection, and co-exposure to multiple pesticide residues within stored pollen and honey are all known to modulate detoxification enzyme activity and immune competence in honeybees and could plausibly exacerbate the seasonal susceptibility patterns identified in this single-stressor laboratory bioassay. Because the present design isolated each insecticide under controlled conditions, the LC_50_ and LC_90_ values reported here should be interpreted as baseline, best-case estimates of intrinsic toxicity rather than as direct predictors of colony-level outcomes under realistic, multi-stressor field exposure, where sublethal immune suppression and pathogen load could lower the effective threshold for mortality well below the values reported in Table 1, Table 2, Table 3 and Table 4.

These findings support incorporating winter-specific toxicity data into pollinator risk assessment frameworks; since this study was conducted under laboratory conditions, field investigations are still needed to confirm these patterns under natural exposure scenarios. Future studies should investigate sublethal and behavioral effects of these insecticides on colony performance and pollination efficiency. Specifically, future research should: (1) extend exposure assessments to semi-field and field conditions to capture realistic multi route exposure pathways (contact, dietary, and via guttation water); (2) examine the molecular and biochemical basis of seasonal susceptibility differences, including detoxification enzyme activity (e.g., cytochrome P450s, esterases, GSTs) in winter versus summer bees; (3) assess the impact of these insecticides on vitellogenin levels and immune gene expression in winter bees, as these are key determinants of overwintering success; and (4) conduct multi-colony studies across diverse geographic and climatic contexts to confirm the generalizability of the seasonal susceptibility patterns documented here. Such studies would bridge the gap between laboratory toxicology and real-world colony health outcomes, providing the mechanistic and ecological evidence needed for evidence-based revision of pesticide risk assessment protocols for pollinators globally. In overall, the current findings highlight that seasonal physiology is a key, yet underappreciated, modulator of honeybee vulnerability to insecticides. For the most part, regulatory testing for acute toxicity is done using summer workers in a standardized laboratory environment, while wintering colonies undergo very different physiology and exposure routes. Thus, considering seasonal dynamics in pesticide risk assessment schemes would enhance the ecological relevance of pesticide toxicity testing and support the development of pollinator-protective pest management strategies.

## 5. Conclusions

Under the conditions of this study, it can be concluded that emamectin benzoate was the most toxic substance and indoxacarb the least toxic. Toxicity increased with exposure time. Winter honeybees showed greater sensitivity than summer honeybees. This highlights the need to incorporate seasonal honeybee physiology into pesticide risk assessment and supports the use of pollinator-safe integrated pest management practices to reduce negative effects on honeybee colonies. Furthermore, a field-relevant Hazard Quotient (HQ) analysis indicated that indoxacarb, despite its comparatively low acute toxicity, exhibited the narrowest margin between field-recommended spray concentrations and lethal thresholds among the three insecticides, underscoring the importance of integrating field-relevant exposure metrics alongside LC_50_-based rankings in pollinator risk assessment. This narrower margin stems mainly from indoxacarb’s higher recommended field application rate relative to its LC_50_, rather than from a greater intrinsic toxicity than the other two compounds, and the two dimensions of risk should therefore be considered separately rather than conflated.

## Figures and Tables

**Table 1:** Toxicity of selected insecticides against winter honeybee workers (*Apis mellifera* L.) after 24 h of oral exposure.

Treatments	Slope ± SE	LC_50_ (FL) 95%	Toxicity Index (LC_50_)	LC_90_ (FL) 95%	Toxicity Index (LC_90_)	Chi-Square (χ^2^)	df
Emamectin benzoate	1.294 ± 0.084	0.035 (0.010–0.122)	100	0.338 (0.102–9.090)	100	38.47	4
Sulfoxaflor	1.317 ± 0.098	1.101 (0.252–3.315)	3.18	10.354 (3.412–373.745)	3.27	20.91	3
Indoxacarb	1.127 ± 0.104	5.720 (2.725–14.994)	0.61	78.458 (24.886–1730.858)	0.43	8.54	3

LC_50_ = Lethal concentration required to kill 50% of the tested honeybees, LC_90_ = Lethal concentration required to kill 90% of the tested honeybees, FL = Fiducial limits at 95% confidence level, Toxicity index = (LC_50_ of the most toxic insecticide/LC_50_ of the tested insecticide) × 100. LC_50_ values were considered significantly different when 95% fiducial limits did not overlap.

**Table 2:** Toxicity of selected insecticides against winter honeybee workers (*Apis mellifera* L.) after 48 h of oral exposure.

Treatments	Slope ± SE	LC_50_ (FL) 95%	Toxicity Index (LC_50_)	LC_90_ (FL) 95%	Toxicity Index (LC_90_)	Chi-Square (χ^2^)	df
Emamectin benzoate	1.600 ± 0.151	0.004 (0.001–0.008)	100	0.023 (0.010–0.262)	100	20.49	4
Sulfoxaflor	2.043 ± 0.207	0.187 (0.148–0.229)	2.14	0.792 (0.608–1.132)	2.90	3.84	3
Indoxacarb	2.144 ± 0.157	0.725 (0.104–9.484)	0.55	2.870 (0.842–14400.578)	0.80	38.91	3

LC_50_ = Lethal concentration required to kill 50% of the tested honeybees, LC_90_ = Lethal concentration required to kill 90% of the tested honeybees, FL = Fiducial limits at 95% confidence level, Toxicity index = (LC_50_ of the most toxic insecticide/LC_50_ of the tested insecticide) × 100. LC_50_ values were considered significantly different when 95% fiducial limits did not overlap.

**Table 3:** Toxicity of selected insecticides against summer honeybee workers (*Apis mellifera* L.) after 24 h of oral exposure.

Treatments	Slope ± SE	LC_50_ (FL) 95%	Toxicity Index (LC_50_)	LC_90_ (FL) 95%	Toxicity Index (LC_90_)	Chi-Square (χ^2^)	df
Emamectin benzoate	1.211 ± 0.096	0.036 (0.004–0.159)	100	1.036 (0.239–45.826)	100	26.60	3
Sulfoxaflor	3.593 ± 0.430	0.958 (0.858–1.117)	3.76	2.009 (1.600–2.874)	51.56	3.59	2
Indoxacarb	2.774 ± 0.512	13.356 (11.933–14.925)	0.27	38.694 (28.800–71.534)	2.68	0.472	2

LC_50_ = Lethal concentration required to kill 50% of the tested honeybees, LC_90_ = Lethal concentration required to kill 90% of the tested honeybees, FL = Fiducial limits at 95% confidence level, Toxicity index = (LC_50_ of the most toxic insecticide/LC_50_ of the tested insecticide) × 100. LC_50_ values were considered significantly different when 95% fiducial limits did not overlap.

**Table 4:** Toxicity of selected insecticides against summer honeybee workers (*Apis mellifera* L.) after 48 h of oral exposure.

Treatments	Slope ± SE	LC_50_ (FL) 95%	Toxicity Index (LC_50_)	LC_90_ (FL) 95%	Toxicity Index (LC_90_)	Chi-Square (χ^2^)	df
Emamectin benzoate	1.305 ± 0.125	0.009 (0.002–0.029)	100	0.134 (0.042–2.438)	100	22.102	3
Sulfoxaflor	3.347 ± 0.371	0.571 (0.521–0.627)	1.58	1.380 (1.156–1.797)	9.71	1.85	2
Indoxacarb	3.558 ± 0.281	4.545 (2.650–6.214)	0.20	10.417 (7.627–17.714)	1.29	10.04	3

LC_50_ = Lethal concentration required to kill 50% of the tested honeybees, LC_90_ = Lethal concentration required to kill 90% of the tested honeybees, FL = Fiducial limits at 95% confidence level, Toxicity index = (LC_50_ of the most toxic insecticide/LC_50_ of the tested insecticide) × 100. LC_50_ values were considered significantly different when 95% fiducial limits did not overlap.

**Table 5:** Comparative Sensitivity Ratio (SR) between Winter and Summer honeybees.

Treatments	Time	Winter LC_50_	Summer LC_50_	Sensitivity Ratio	More Sensitive Season
Emamectin benzoate	24	0.035	0.036	1.03	winter
Emamectin benzoate	48	0.004	0.009	2.25	winter
Sulfoxaflor	24	1.101	0.958	1.15	summer
Sulfoxaflor	48	0.187	0.571	3.05	winter
Indoxacarb	24	5.720	13.356	2.34	winter
Indoxacarb	48	0.725	4.545	6.26	winter

Sensitivity Ratio (SR) = LC_50_ of the less sensitive season/LC_50_ of the more sensitive season, SR values > 1 indicate higher susceptibility of the more sensitive season.

**Table 6:** Toxicity Increase Factor (TIF) over exposure duration (24 h vs. 48 h).

Treatments	Season	24 h LC_50_	48 h LC_50_	Toxicity Increase Factor
Emamectin benzoate	winter	0.035	0.004	8.75×
Sulfoxaflor	winter	1.101	0.187	5.88×
Indoxacarb	winter	5.720	0.725	7.88×
Emamectin benzoate	summer	0.036	0.009	4.00×
Sulfoxaflor	summer	0.958	0.571	1.67×
Indoxacarb	summer	13.356	4.545	2.93×

Toxicity Increase Factor (TIF) = LC_50_ at 24 h/LC_50_ at 48 h, Higher TIF values indicate greater time-dependent increase in toxicity, × = fold increase in toxicity over exposure time.

**Table 7:** Field-relevant Hazard Quotient (HQ) of selected insecticides, calculated from oral LC_50_ values and official field-recommended spray concentrations.

Treatments	Time	Season	Field-Recommended Concentration (mg a.i./L)	LC_50_ (mg/L)	Hazard Quotient (HQ)
Emamectin benzoate	24	Winter	23.75	0.035	678.57
Emamectin benzoate	48	Winter	23.75	0.004	5937.50
Emamectin benzoate	24	Summer	23.75	0.036	659.72
Emamectin benzoate	48	Summer	23.75	0.009	2638.89
Sulfoxaflor	24	Winter	58.5	1.101	53.13
Sulfoxaflor	48	Winter	58.5	0.187	312.83
Sulfoxaflor	24	Summer	58.5	0.958	61.06
Sulfoxaflor	48	Summer	58.5	0.571	102.45
Indoxacarb	24	Winter	90	5.720	15.73
Indoxacarb	48	Winter	90	0.725	124.14
Indoxacarb	24	Summer	90	13.356	6.74
Indoxacarb	48	Summer	90	4.545	19.80

HQ = Field-recommended spray concentration (mg a.i./L)/LC_50_ (mg/L). Higher HQ values indicate a wider margin between the field-recommended spray concentration and the experimentally derived lethal threshold. Field-recommended concentrations were calculated from the manufacturer’s label dosage: Basha 1.9% EC (emamectin benzoate) at 1.25 mL/L water; Isoclast 11.7% SC (sulfoxaflor) at 0.50 mL/L water; Easo Plus 30% WG (indoxacarb) at 0.3 g/L water.

## Data Availability

The raw data supporting the conclusions of this article will be made available by the authors on request.
